# Identification and validation of key host genes associated with porcine H1N1 infection based on integrated machine learning algorithms

**DOI:** 10.3389/fvets.2026.1906560

**Published:** 2026-08-12

**Authors:** YanNa Guo, JinTao Liu, ZiLong He, XuDong Han, HeYun Yang, Hua Zhang, PanPan Sun, KuoHai Fan, Wei Yin, Jia Zhong, ZhenBiao Zhang, HuiZhen Yang, JianZhong Wang, YaoGui Sun, ShaoYu Wang, HongQuan Li, Na Sun

**Affiliations:** 1 College of Veterinary Medicine, Shanxi Key Laboratory of Modernization of Traditional Chinese Veterinary Medicine, Shanxi Agricultural University, Shanxi, China; 2 Baoding Jizhong Pharmaceutical Co., Ltd., Baoding, Hebei, China; 3 Laboratory Animal Management Center, Shanxi Key Laboratory of Modernization of Traditional Chinese Veterinary Medicine, Shanxi Agricultural University, Jinzhong, Shanxi, China; 4 School of Dentistry and Medical Sciences, Charles Sturt University, Orange, NSW, Australia

**Keywords:** H1N1 influenza virus, innate immunity, machine learning, piglets, SPP1

## Abstract

**Objective:**

Swine H1N1 influenza is a critical zoonotic pathogen threatening pig industry economy and public health. The host molecular regulatory network and core genes of H1N1 infection remain unclear, hindering targeted prevention and therapy. Traditional experimental methods fail to efficiently mine high-dimensional transcriptomic data, making precise screening of infection biomarkers difficult. Methods: Transcriptome data (GSE40092) were analyzed to obtain porcine lung DEGs upon H1N1 infection, followed by GO/KEGG functional enrichment. Four machine learning algorithms (LASSO, random forest, SVM-RFE, XGBoost) coupled with stratified nested 5-fold cross-validation screened core genes. Feature stability analysis and external dataset GSE28871 validated biomarker robustness. A gradient-dose H1N1 piglet model and Western blot verified the key gene’s *in vivo* protein expression.

**Results:**

A total of 310 H1N1-related DEGs were enriched in immune, inflammatory and viral signaling pathways. All four models accurately discriminated infected and normal lung samples, with SPP1 as the only shared core gene. Cross-validation proved SPP1 screening free of overfitting; external validation yielded an AUC of 0.889, 83.3% sensitivity and 100% specificity. *In vivo* assays confirmed significant SPP1 protein downregulation under low, medium and high viral doses (*p* < 0.05).

**Conclusion:**

This study combined transcriptomics and multi-machine learning to identify and verify host genes for swine H1N1 infection. SPP1 acts as a stable diagnostic biomarker whose reduced expression correlates with disease progression. Our results reveal new molecular mechanisms of H1N1 pathogenesis and offer a candidate target for swine flu control and zoonotic risk intervention.

## Graphical abstract

**Figure:**
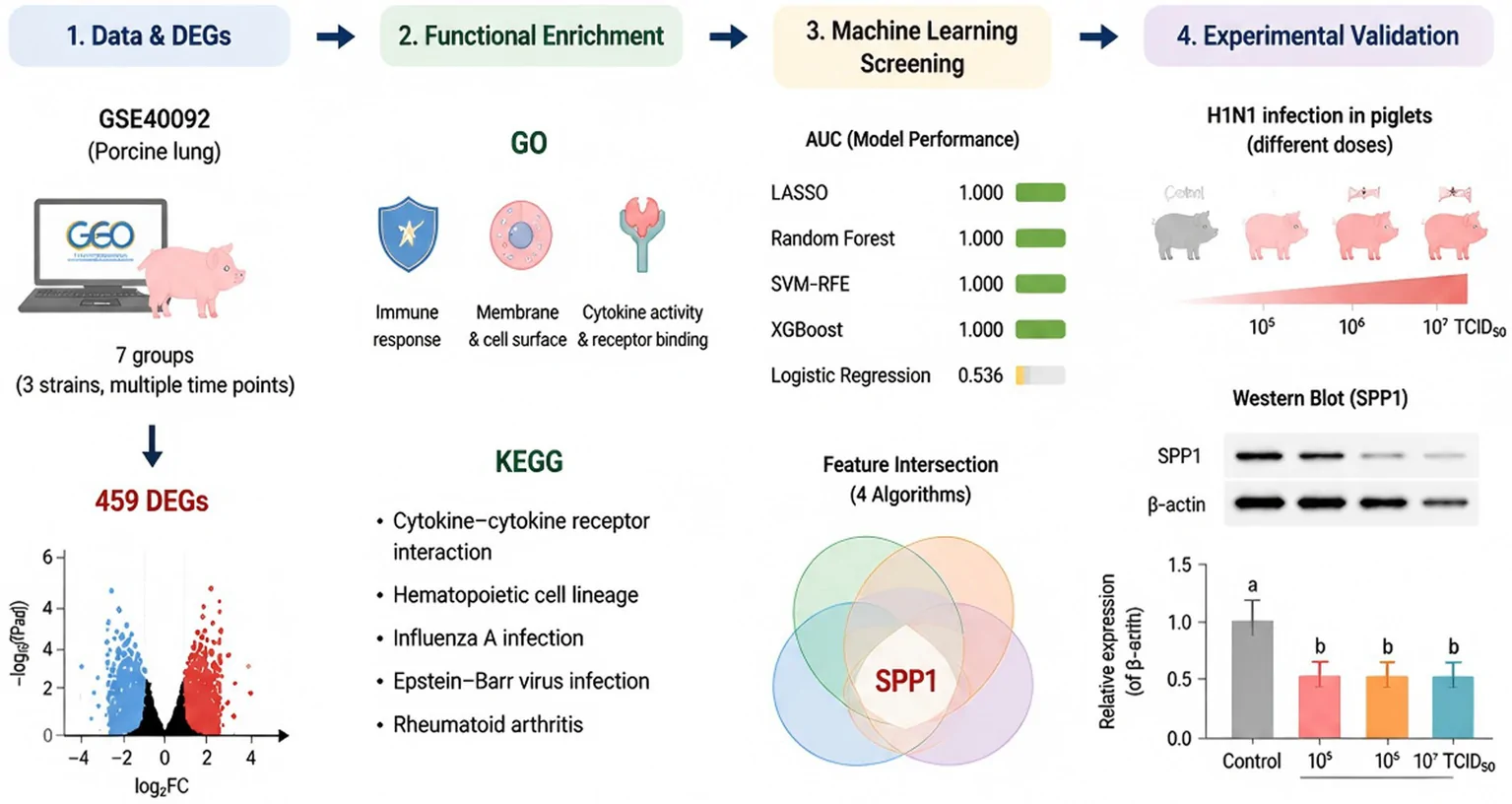

## 1 Introduction

Influenza virus is a major zoonotic pathogen worldwide that poses a serious threat to both human and animal health. Among its subtypes, H1N1 has garnered considerable attention due to its high mutation rate and strong potential for cross-species transmission (1, 2). H1N1 is classified within the influenza A virus family and is a segmented negative-sense RNA virus primarily transmitted through the respiratory tract. In swine populations, H1N1 infection is characterized primarily by fever, coughing, dyspnea, and reduced growth performance, resulting in substantial economic losses for the pig industry. Moreover, as a zoonotic pathogen, the virus can be efficiently transmitted via aerosols and therefore represents a potential threat to public health security (3). Consequently, elucidating the pathogenic mechanisms of H1N1 infection and identifying its core host targets have become critical scientific issues in influenza prevention and control research.

Traditional biological approaches for identifying disease-associated genes are often time-consuming, inefficient, and are inadequate for analyzing high-dimensional and highly complex omics datasets (4, 5). With the rapid development of high-throughput sequencing technologies, large-scale gene expression data have accumulated continuously. Differentially expressed genes (DEGs) identified from transcriptomic datasets usually include not only genes directly involved in disease progression, but also numerous indirectly responsive genes and noise signals, which substantially increases the difficulty of accurately identifying core targets (6).

Against this backdrop, computational biology approaches, particularly machine learning technologies, have yielded novel strategies for accelerating disease mechanism exploration and key target gene identification (7). Through dimensionality reduction, feature selection, and pattern recognition, machine learning methods can effectively extract discriminative information from complex high-dimensional datasets, thereby improving the accuracy and efficiency of target screening and providing a solid foundation for subsequent functional validation and targeted intervention studies (8).

Therefore, in the present study, transcriptomic data from H1N1-infected piglets were systematically integrated to identify DEGs, followed by functional enrichment analyses to elucidate the key biological processes and signaling pathways involved. Subsequently, multiple machine learning algorithms, including LASSO regression, random forest, support vector machine recursive feature elimination (SVM-RFE), and eXtreme Gradient Boosting (XGBoost), were comprehensively applied for multidimensional screening and cross-validation of candidate genes, to accurately identify key host target genes involved in H1N1 infection. Furthermore, piglet models infected with different doses of H1N1 virus were established, and the expression of key genes was validated at the protein level by Western blot analysis. Through this multi-level integrative analysis, this study seeks to clarify the molecular regulatory mechanisms underlying H1N1 infection and provide potential therapeutic targets and theoretical support for the development of precise prevention and control of swine influenza.

## 2 Materials and methods

### 2.1 Virus, cells, and major reagents

Madin–Darby canine kidney (MDCK) cells were passaged routinely and preserved in liquid nitrogen. The H1N1 influenza virus strain (CVCC AV1522) was purchased from the China Veterinary Culture Collection Center (CVCC). The viral propagation was carried out in MDCK cells, and the viral titer was determined to be 10^7.83^ TCID₅₀/0.1 mL using the Reed–Muench method.

DMEM liquid medium (C119955OOBT) was purchased from Gibco; fetal bovine serum (SA211.02) was obtained from Cellmax; trypsin (T917515) and penicillin–streptomycin solution (P917928) were purchased from Macklin. Goat anti-rabbit IgG-HRP antibody (abs20040) was purchased from Absin; Osteopontin recombinant rabbit monoclonal antibody (bsm-43600R) was obtained from Bioss; and *β*-actin antibody (13E5) was purchased from Cell Signaling Technology.

### 2.2 GEO data acquisition and preprocessing

The porcine lung transcriptomic dataset following H1N1 infection, GSE40092, was retrieved from the GEO database of the National Center for Biotechnology Information (NCBI) (GEO Database). This dataset comprised a total of 56 target samples, including lung tissue samples collected from piglets infected with the H1N1 strains A/swine/Iowa/15/1930 (designated as 1930IA15) at 3 and 5 days post-infection (dpi), A/swine/Alberta/25/2009 (designated as ALB) at 3 and 5 dpi, and A/Ca/04/2009 (designated as ca04) at 3, 5, and 7 dpi, together with corresponding control lung tissue samples.

Differential expression analysis was performed using the “Analyze with GEO2R” tool provided by NCBI. Differentially expressed genes (DEGs) between H1N1-infected and control piglets were screened using the criteria of |log2 fold change (FC)| > 0.26 and adjusted *p* < 0.05, and these DEGs were subsequently used as the candidate feature set for further analysis.

### 2.3 Functional and pathway enrichment analyses of Core target genes

Functional enrichment analysis of the identified core target genes was performed using the DAVID Database. Gene Ontology (GO) enrichment analysis and Kyoto Encyclopedia of Genes and Genomes (KEGG) pathway enrichment analyses were conducted to investigate the biological significance of these genes. By analyzing the enriched biological processes, cellular components, molecular functions, and significantly associated signaling pathways, the potential molecular mechanisms of key host genes involved in porcine H1N1 virus infection were delineated.

### 2.4 Identification of feature genes and construction of machine learning models

In this study, four machine learning algorithms, including LASSO regression, random forest (RF), support vector machine (SVM), and eXtreme Gradient Boosting (XGBoost), were comprehensively applied for high-dimensional data reduction and key feature selection based on the previously identified differentially expressed genes (DEGs). The DEG expression matrix was used as the input dataset, and the machine learning models were constructed and trained using the Python (scikit-learn + XGBoost).

For the LASSO regression model, the optimal regularization parameter (*λ*) was determined through L1-penalized logistic regression with grid-search optimization (C ∈ {0.001, 0.01, 0.1, 0.5, 1.0, 5.0, 10.0}) under the inner cross-validation loop. In the random forest model, the number of decision trees (ntree) was set to 200 with a maximum tree depth of 5 to achieve a balance between computational efficiency and model stability. For the XGBoost model, the learning rate (eta) was set to 0.01 with a maximum tree depth of 3 and 100 boosting rounds, incorporating L1 (*α* = 0.5) and L2 (*λ* = 1.0) regularization terms to control overfitting. For the SVM model, a radial basis function (RBF) kernel was employed with grid-searched C and *γ* parameters.

A stratified nested 5-fold cross-validation framework was applied to achieve unbiased model evaluation and avoid data leakage. Briefly, the outer 5-fold loop was used for model performance assessment, while the inner 5-fold loop was dedicated to hyperparameter optimization. Stratified sampling was performed to maintain consistent case–control ratios across all folds based on the entire dataset containing 56 samples. To eliminate information leakage, data standardization (*Z*-score) was strictly trained only on the outer training set during each iteration. All 310 differentially expressed genes (adjusted *p* < 0.05, |log₂FC| > 0.26) identified from the seven subgroup comparisons were adopted as input features. Four classification models, including LASSO logistic regression, random forest, support vector machine, and XGBoost, were constructed with grid-search optimized hyperparameters. Model diagnostic performance was quantified by the average AUC values from the outer cross-validation folds. Gene feature importance scores were independently calculated by each algorithm, and key feature genes were identified stepwise from different models. In conclusion, the intersection of feature genes identified by the four machine learning algorithms was defined as the core target gene set capable of effectively distinguishing H1N1-infected samples from control samples.

### 2.5 Feature stability validation via nested cross-validation

To verify that the selection of SPP1 as a candidate biomarker was not driven by overfitting to a single data partition, we evaluated the cross-fold stability of feature importance rankings within the nested cross-validation framework. For each of the 5 outer folds, the optimal model (selected by the inner 5-fold CV loop) was trained on the outer training set, and the top 20 features ranked by importance were recorded. This process was repeated independently for all four ML algorithms (LASSO, Random Forest, SVM, and XGBoost), yielding 20 independent feature rankings (4 models × 5 outer folds).

Feature importance was defined as follows for each algorithm:

LASSO: absolute value of the L1-regularized logistic regression coefficients Random Forest: Gini impurity-based feature importance (feature_importances_) SVM: absolute value of the linear kernel coefficients XGBoost: gain-based feature importance (feature_importances_) For each gene, we computed its cross-fold frequency — the number of times (out of 20 evaluations) it appeared among the top 20 features. A gene with high cross-fold frequency indicates that its predictive contribution is consistent across different data partitions and modeling approaches, providing strong evidence against overfitting. Conversely, a gene that appears only in a single fold or model is likely a spurious selection driven by that specific data split.

### 2.6 Validation of SPP1 as a biomarker using an independent dataset

To verify the robustness of SPP1 as a biomarker, an independent external dataset was used for validation in this study. The porcine lung transcriptomic dataset following H1N1 infection, GSE28871, was retrieved from the GEO database of the National Center for Biotechnology Information (NCBI) (GEO Database). This dataset comprised a total of 12 lung tissue samples in total, consisting of piglets challenged with the H1N1 strain A/swine/Hubei/101/2009 at 3 and 7 days post infection (dpi), alongside corresponding mock-infected control groups matched to each sampling time point.

### 2.7 Establishment of the piglet H1N1 infection model and target validation

#### 2.7.1 Establishment of the H1N1-infected piglet model

Twelve healthy weaned piglets (28 days old) were purchased from Jinzhong Xuhong Livestock Breeding Service Co., Ltd. and acclimated for 7 days prior to experimentation. The piglets were then randomly divided into four groups (*n* = 3 per group): a control group, a high-dose H1N1 infection group (10^7^ TCID₅₀), a medium-dose H1N1 infection group (10^6^ TCID₅₀), and a low-dose H1N1 infection group (10^5^ TCID₅₀).

Following acclimation, piglets in the infection groups were intranasally inoculated with 2 mL of H1N1 virus suspension (1 mL per nostril), whereas piglets in the control group received the same volume of DMEM medium via the same route. On day 8 post-infection, all piglets were euthanized [Pigs were euthanized with sodium pentobarbital (150 mg/kg, i.v.). Death was confirmed by the absence of respiration, heartbeat, and corneal reflex. Tissues were collected immediately thereafter], and lung tissues were aseptically collected, immediately snap-frozen in liquid nitrogen, and subsequently stored at −80 °C for further analysis.

#### 2.7.2 Detection of SPP1 protein expression by Western blot

A protease and phosphatase inhibitor cocktail was mixed with RIPA lysis buffer at a ratio of 1:100 for the extraction of total proteins from piglet lung tissues. Protein samples were separated by SDS–PAGE and subsequently transferred onto 0.22 μm PVDF membranes using a wet transfer system under low-temperature conditions. The membranes were blocked with 5% nonfat milk at room temperature for 2 h and washed three times with TBST.

The PVDF membranes were then incubated overnight at 4 °C with primary antibodies against SPP1 and *β*-actin, respectively. After incubation, the membranes were washed three times with TBST for 10 min each. Subsequently, HRP-conjugated goat anti-rabbit or goat anti-mouse secondary antibodies were applied and incubated at room temperature for 1 h, followed by three additional washes with TBST for 10 min each. Protein bands were visualized using enhanced chemiluminescence (ECL) reagent, and grayscale intensities of the bands were quantified using ImageJ software.

### 2.8 Statistical analysis

All experimental data were expressed as mean ± standard deviation (Mean ± SD). Statistical analyses were performed using GraphPad Prism software (GraphPad Software Inc., California, United States). Before statistical comparisons, data normality was assessed using the Shapiro–Wilk test, and homogeneity of variance was evaluated by Levene’s test. Data satisfying the assumptions of normal distribution and homogeneity of variance were analyzed using one-way analysis of variance (one-way ANOVA) followed by Tukey’s multiple comparison test. A value of *p* < 0.05 was considered statistically significant, and significant differences among groups were indicated by different lowercase letters as superscripts.

## 3 Results

### 3.1 Differentially expressed genes

**Figure 1:**
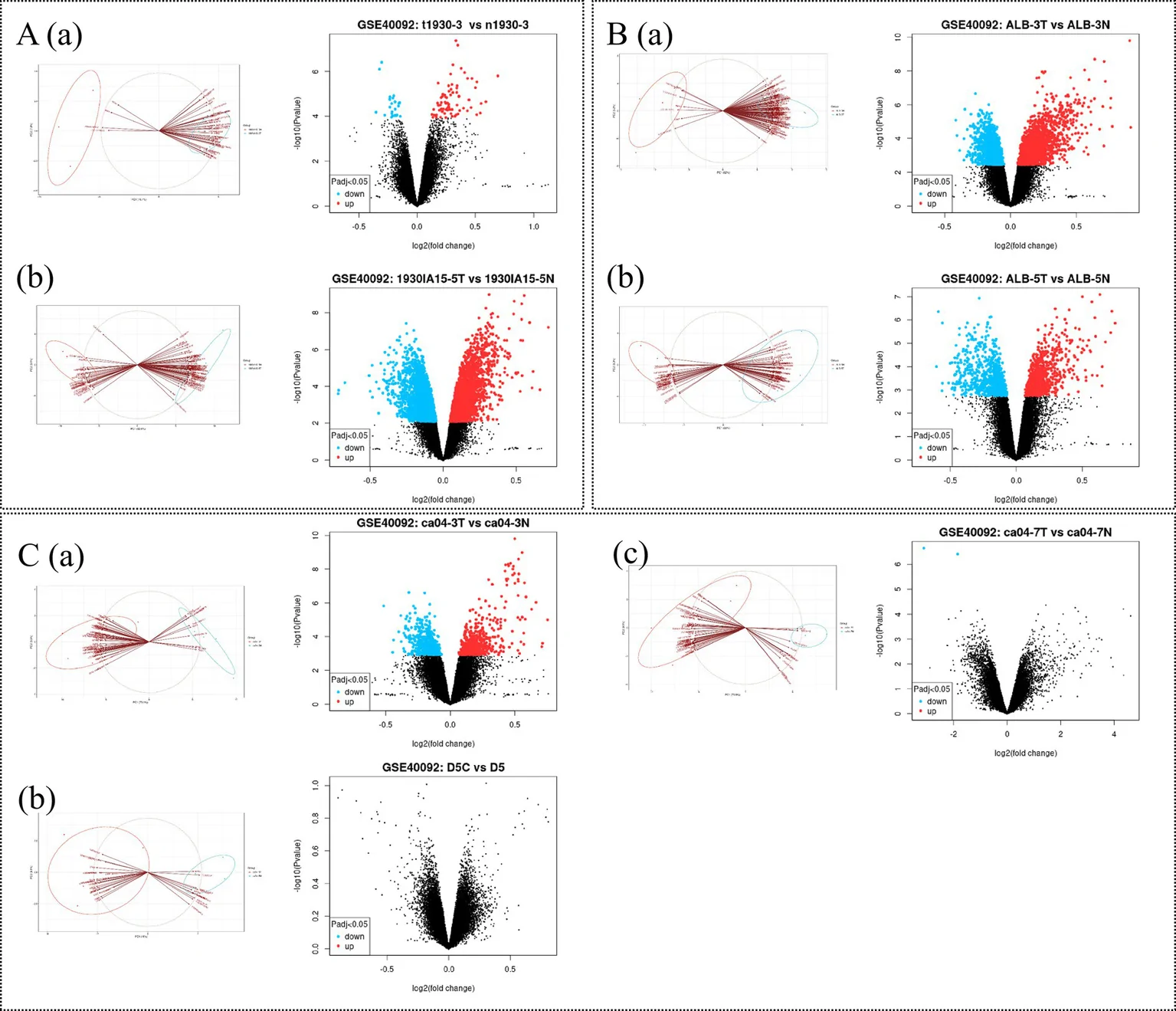
PCA and volcano plot analyses of differentially expressed genes in the GSE40092 dataset. **(A–C)** Transcriptomic comparisons of piglets infected with three different H1N1 virus strains. **(A)** 1930IA15 strain: (a) 3 days post-infection vs. control; (b) 5 days post-infection vs. control. **(B)** ALB strain: (a) 3 days post-infection vs. control; (b) 5 days post-infection vs. control. **(C)** CA04 strain: (a) 3 days post-infection vs. control; (b) 5 days post-infection vs. control; (c) 7 days post-infection vs. control.

Based on different viral strains and infection time points, the GSE40092 dataset was divided into seven experimental groups, including 1930IA15-3, 1930IA15-5, ALB-3, ALB-5, ca04-3, ca04-5, and ca04-7. A total of 310 differentially expressed genes (DEGs) were identified (Figure 1).

Principal component analysis (PCA) demonstrated a clear separation in the overall expression profiles between infected and control samples in the 1930IA15-3, 1930IA15-5, ALB-3, ALB-5, and ca04-3 groups, indicating good intragroup reproducibility. In contrast, the ca04-5 and ca04-7 groups showed relatively weak separation from the control samples.

Volcano plot analysis further confirmed that a large number of DEGs were identified in the significantly differentiated groups. In the volcano plots, red dots represent significantly upregulated genes, blue dots represent significantly downregulated genes (|log₂FC| > 0.26, adjusted *p* < 0.05), and black dots indicate genes without significant differential expression. Moreover, the numbers of DEGs in the 1930IA15 and ALB infection groups increased with prolonged infection duration, suggesting that H1N1 infection induced extensive transcriptomic responses in a time-dependent manner. In contrast, the numbers of DEGs in the ca04 strain infection groups decreased at 5 and 7 days post-infection.

### 3.2 Functional enrichment analysis

**Figure 2:**
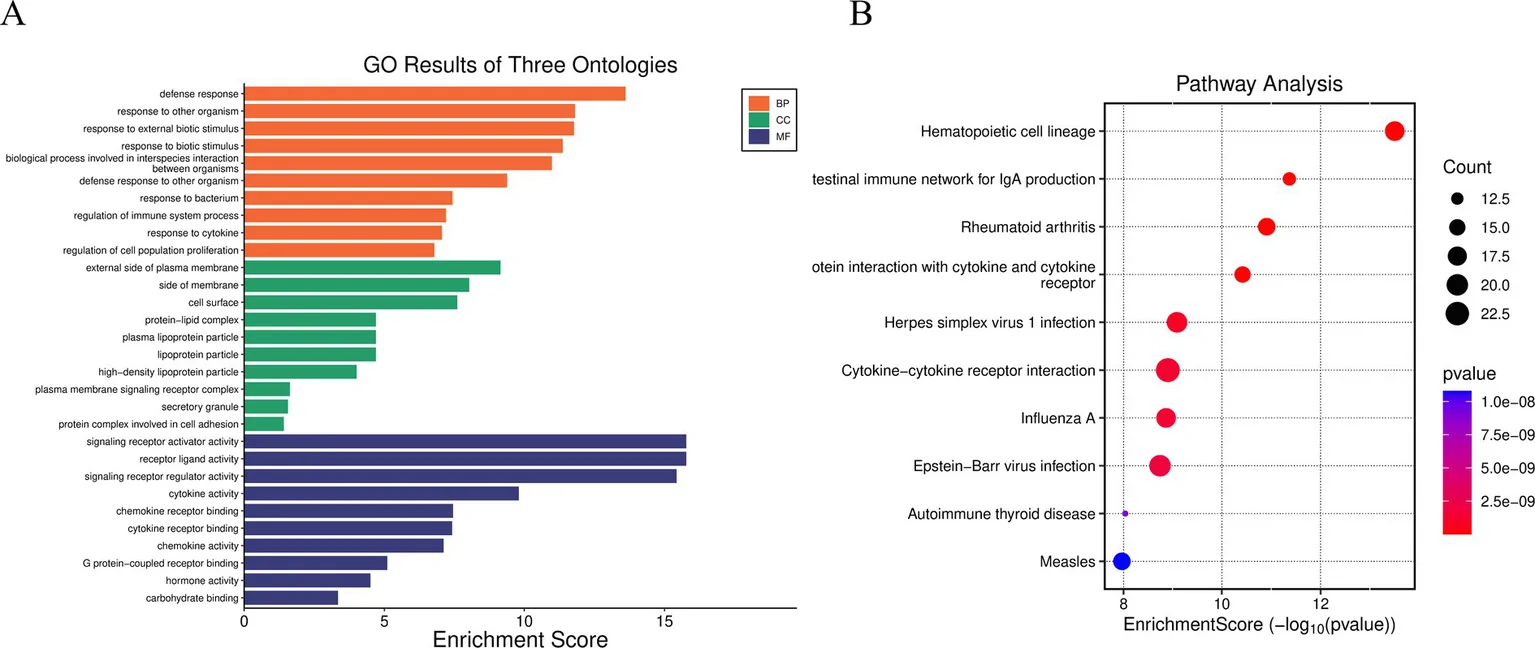
GO and KEGG enrichment analyses of differentially expressed genes. **(A)** GO enrichment analysis results. **(B)** KEGG pathway enrichment analysis results.

Gene Ontology (GO) enrichment analysis (Figure 2A) revealed that, at the biological process (BP) level, the differentially expressed genes (DEGs) were mainly enriched in defense response, response to biotic stimulus, immune response, and cytokine-related biological processes. At the cellular component (CC) level, the DEGs were primarily enriched in the plasma membrane, cell surface, and protein–lipid complexes. At the molecular function (MF) level, significant enrichment was observed in signaling receptor ligand activity, cytokine activity, and chemokine receptor binding.

KEGG pathway enrichment analysis (Figure 2B) demonstrated that the DEGs were significantly enriched in cytokine–cytokine receptor interaction, hematopoietic cell lineage, and several virus infection-related pathways, including influenza A, Epstein–Barr virus infection, and herpes simplex virus infection, as well as inflammatory and immune-related pathways such as rheumatoid arthritis. These findings indicate that the identified DEGs are mainly involved in immune regulation, inflammatory responses, and host antiviral response processes.

### 3.3 Machine learning analysis

**Figure 3:**
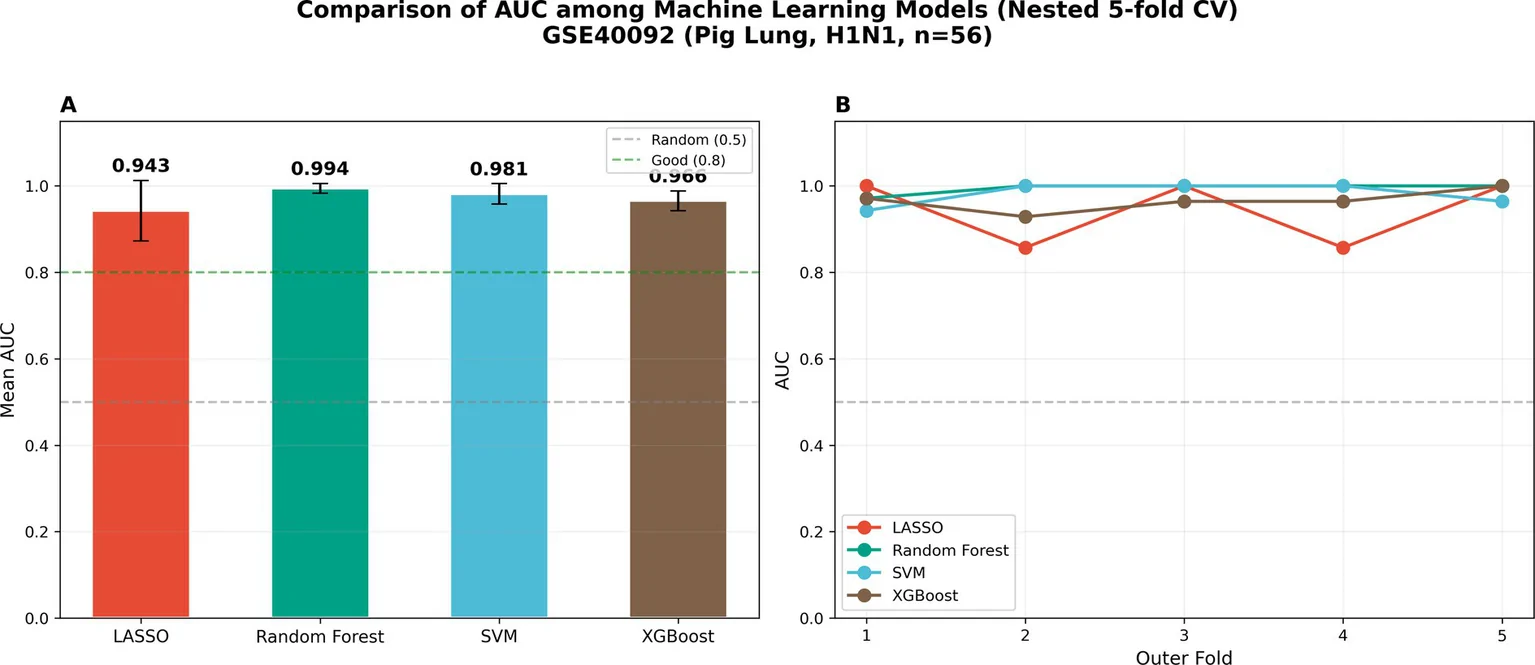
Comparison of the area under the receiver operating characteristic curve (AUC) among four machine learning models. **(A)** Bar plot showing the mean AUC values of LASSO, Random Forest, SVM and XGBoost models. The grey dashed line represents random classification level (AUC = 0.5), and the green dashed line represents good classification performance (AUC = 0.8). Error bars indicate standard deviation. **(B)** Line plot displaying AUC values across five outer cross‑validation folds for the four machine‑learning models.

Stratified nested 5-fold cross-validation was performed to quantify the discriminative capacity of four machine learning algorithms, namely LASSO, random forest, support vector machine and XGBoost, for infected samples, based on classification models constructed from differentially expressed genes derived from the GSE40092 transcriptomic dataset containing 56 porcine lung tissue samples (Figure 3B). The mean area under the curve (AUC) values of the four models, sorted from highest to lowest, were 0.994 for random forest, 0.981 for support vector machine, 0.966 for XGBoost and 0.943 for LASSO. All values were markedly higher than the reliable classification threshold of 0.8 and the random discrimination baseline of 0.5, verifying that every model could effectively distinguish lung tissue samples from H1N1-infected piglets and healthy piglets. Analysis of the fold-wise discriminative performance fluctuations across five outer data partitions revealed that random forest and support vector machine maintained AUC values steadily close to 1.0 throughout all splits with minimal variation; XGBoost only exhibited slight fluctuations and sustained AUC values above 0.94 in each partition; the LASSO model showed relatively weaker stability, with obvious performance declines at the second and fourth folds where its AUC dropped to approximately 0.85, yet it still maintained acceptable sample discriminative power across all independent data partitions. Collectively, these results confirmed that the integrated multi-algorithm machine learning strategy achieved excellent and stable classification performance for porcine H1N1-infected samples under nested cross-validation.

### 3.4 Identification of key feature genes by machine learning algorithms

**Figure 4:**
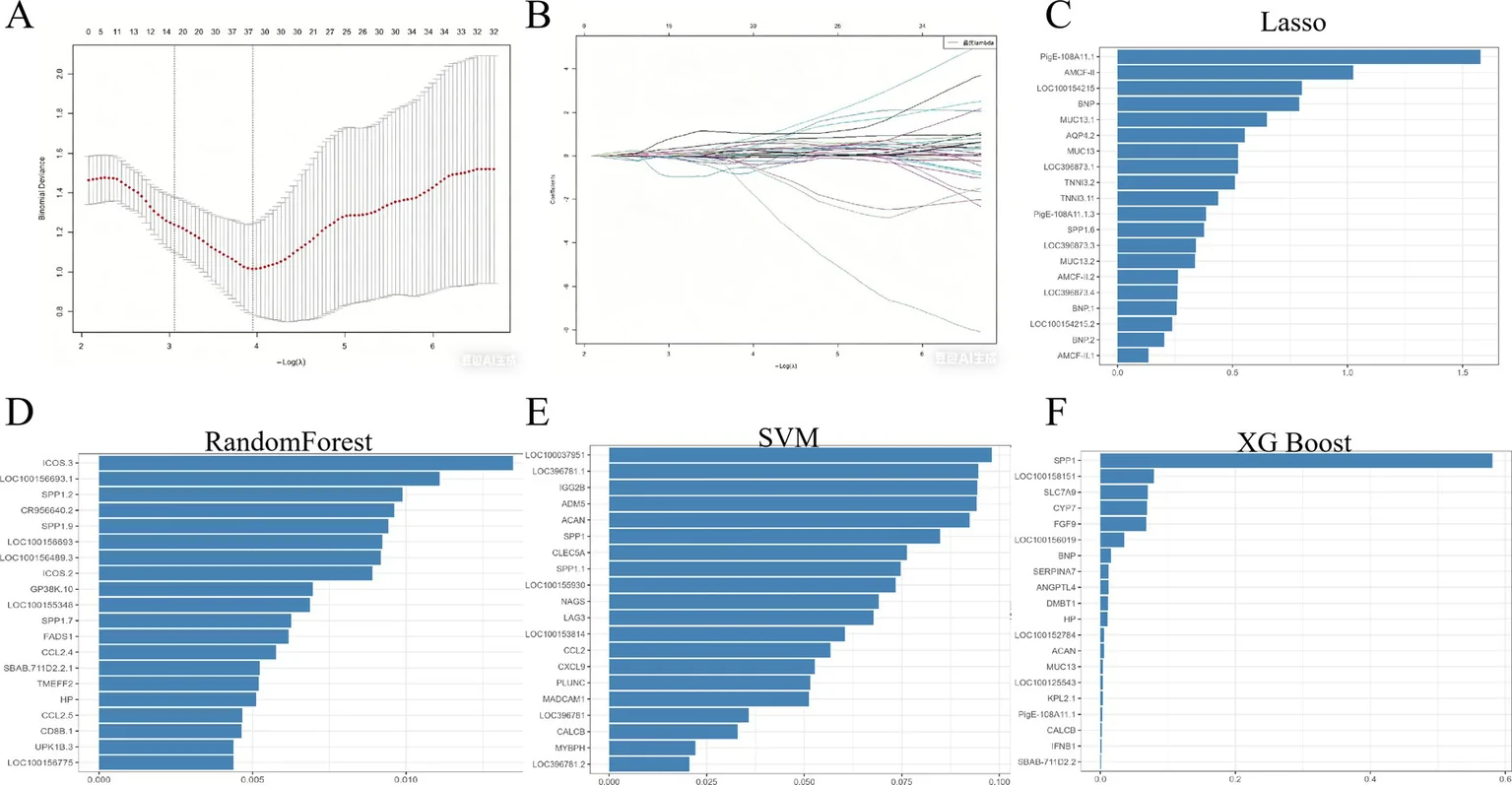
Identification of key feature genes using four machine learning algorithms. **(A)** Five-fold cross-validation error curve. **(B)** LASSO coefficient profile plot. **(C)** Key genes identified by the LASSO regression algorithm. **(D)** Key genes identified by the random forest algorithm. **(E)** Key genes identified by the SVM-RFE algorithm. **(F)** Key genes identified by the XGBoost algorithm and their corresponding importance rankings.

Differentially expressed genes (DEGs) were screened using four machine learning algorithms, including LASSO regression, random forest, SVM, and XGBoost. Among them, the LASSO regression model (Figures 4A–C) demonstrated the optimal *λ* value through cross-validation and identified 19 key feature genes. The random forest (Figure 4D), SVM (Figure 4E), and XGBoost (Figure 4F) models selected the top 20 genes based on feature importance ranking.

Partial overlap was observed among the feature genes identified by different algorithms, collectively forming a set of disease-related key genes. These overlapping genes may serve as core biomarkers for subsequent model construction and experimental validation.

### 3.5 Intersection analysis of different machine learning algorithms

An intersection analysis was carried out on the feature genes identified by the four machine learning algorithms, including LASSO, random forest, SVM, and XGBoost. The Venn diagram analysis revealed that SPP1 was the only key gene consistently identified by all four algorithms.

**Figure 5:**
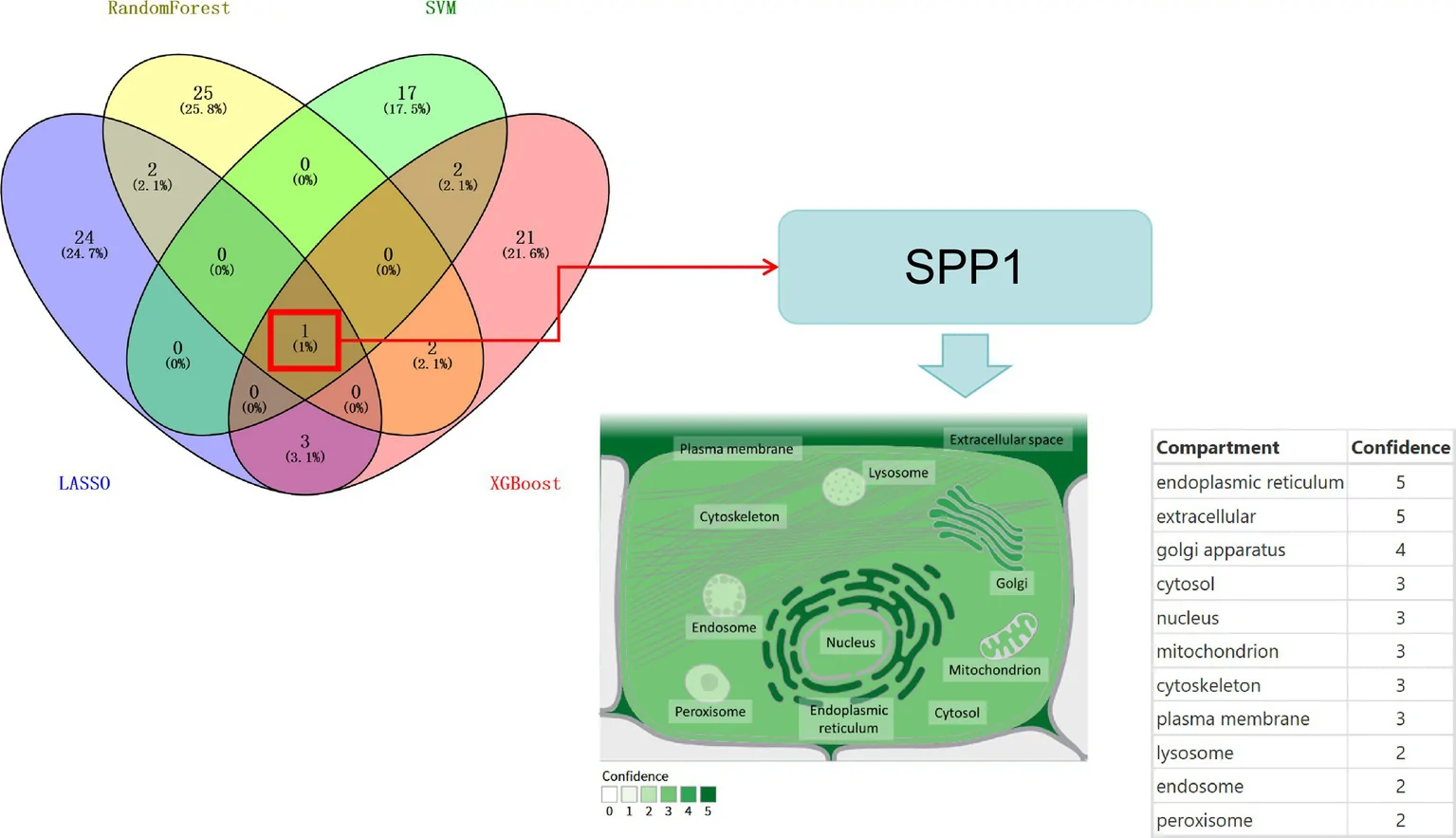
Intersection analysis of feature genes identified by four machine learning algorithms and prediction of the subcellular localization of SPP1.

Subcellular localization analysis demonstrated that SPP1 was primarily localized in the extracellular space and endoplasmic reticulum, while also being distributed in the Golgi apparatus, cytoskeleton, and plasma membrane (Figure 5). Taken together, these findings suggest that SPP1 may participate in extracellular signal regulation through secretory pathways and could serve as a core candidate biomarker for subsequent mechanistic studies.

### 3.6 Feature stability validation via nested cross-validation

**Figure 6:**
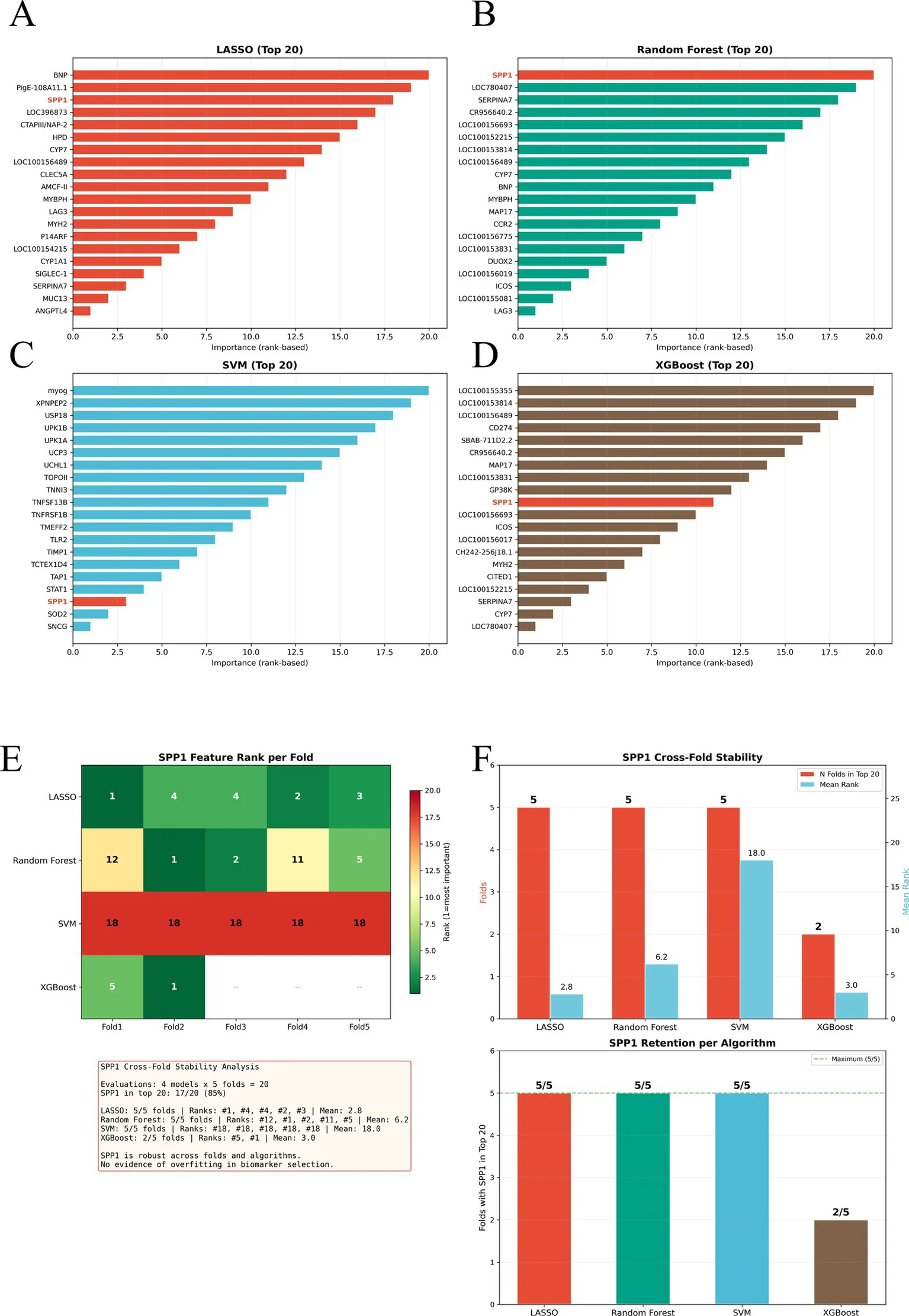
Stability evaluation of SPP1 feature importance via nested cross-validation. **(A–D)** fold-wise selection frequency of top-20 genes under LASSO, Random Forest, SVM and XGBoost models. **(E)** Heatmap displaying SPP1’s feature rank in each outer cross-validation fold. **(F)** Summary bar graph presenting the number of folds retaining SPP1 and its mean rank per algorithm.

We evaluated the screening stability of SPP1 as a candidate biomarker using stratified nested 5-fold cross-validation, where we recorded the top 20 feature genes ranked by importance from LASSO, random forest, support vector machine and XGBoost across five outer data partitions. Figures 6A–D separately illustrate the rank-based feature importance of all genes derived from LASSO, random forest, SVM and XGBoost models, showing that SPP1 ranks high in the first three algorithms while it occupies a lower position in SVM. Figure 6E is a heatmap visualizing the exact feature rank of SPP1 within each fold for every algorithm, and Figure 6F quantifies cross-fold retention frequency and average feature rank: SPP1 is retained in the top 20 features across all five folds for LASSO, random forest and SVM with average ranks of 2.8, 6.2 and 18.0 separately, whereas it only appears in 2 out of 5 folds under XGBoost with an average rank of 3.0. Collectively, LASSO and random forest exhibit outstanding stability in identifying SPP1 as a high-contribution predictive biomarker independent of data splitting, and the consistent detection of SPP1 across most independent screening iterations eliminates the possibility that this gene signature arises from overfitting or random data partition bias.

### 3.7 Validation of SPP1 as a biomarker using an independent dataset

**Figure 7:**
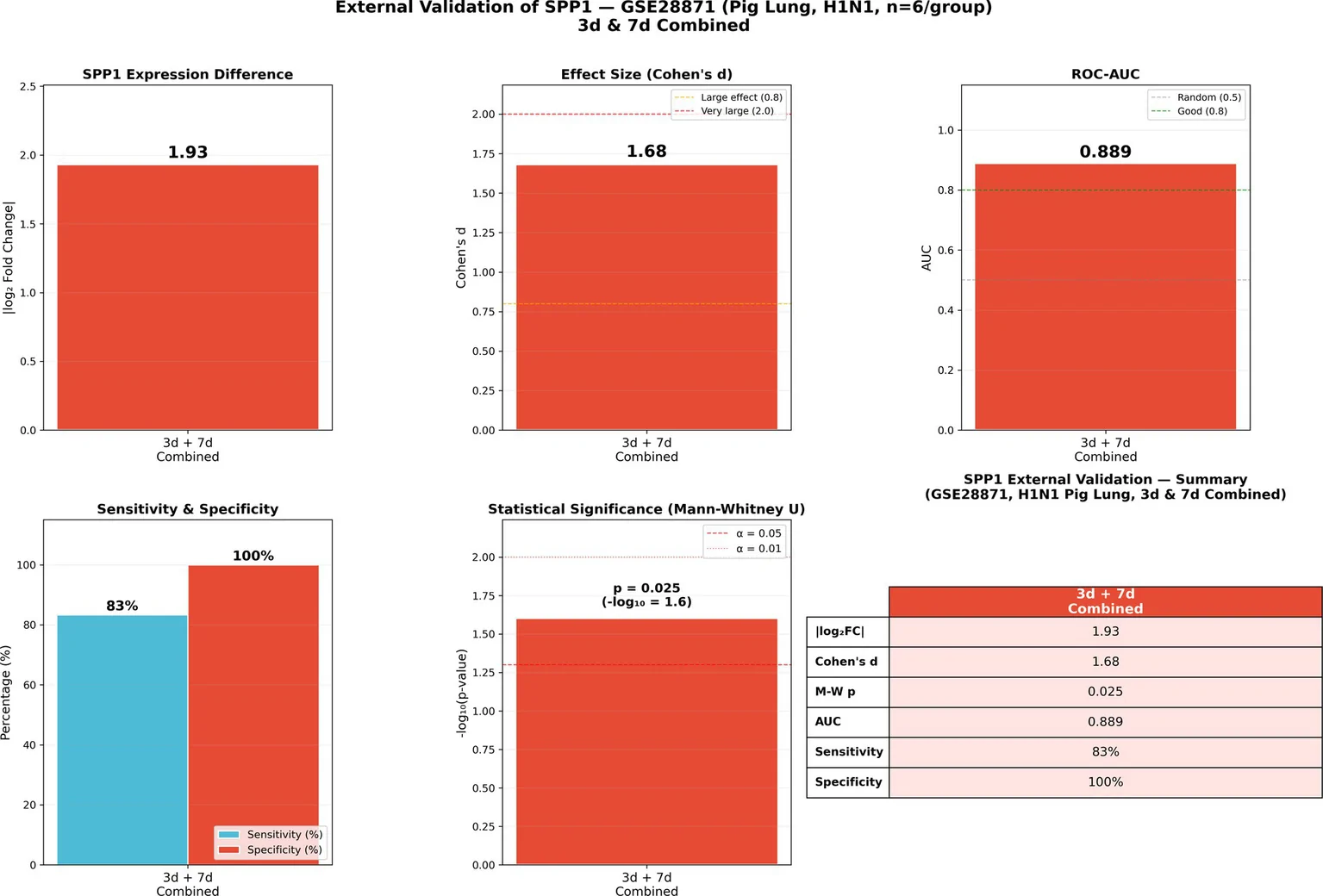
Comprehensive validation of SPP1 for H1N1 discrimination in pooled 3 d and 7 d pig lung samples.

We combined lung samples collected at 3 and 7 days post infection from dataset GSE28871, including six control samples and six H1N1-infected samples, to plot the ROC curve for SPP1. The area under the curve (AUC) reached 0.889 (Figures 7), clearly outperforming the random classification baseline of 0.5, which indicates strong diagnostic power to separate H1N1-infected pigs from healthy ones. Based on the optimal cut-off value derived from the curve, the sensitivity was 83.3% while specificity hit 100%. This translates to a false positive rate of zero: none of the healthy control samples were incorrectly classified as infected, with only a small fraction of infected samples missed during detection. We also observed clear expression differences between the two groups. The absolute log2 fold change was 1.93, accompanied by a large effect size (absolute Cohen’s d = 1.68). The Mann–Whitney *U* test returned a *p* value of 0.025, confirming statistically significant shifts in SPP1 expression across groups. Collectively, these results support that SPP1 could serve as a promising diagnostic biomarker to distinguish H1N1 infection from uninfected status.

### 3.8 Changes in SPP1 protein expression in piglets following H1N1 infection at different doses

**Figure 8:**
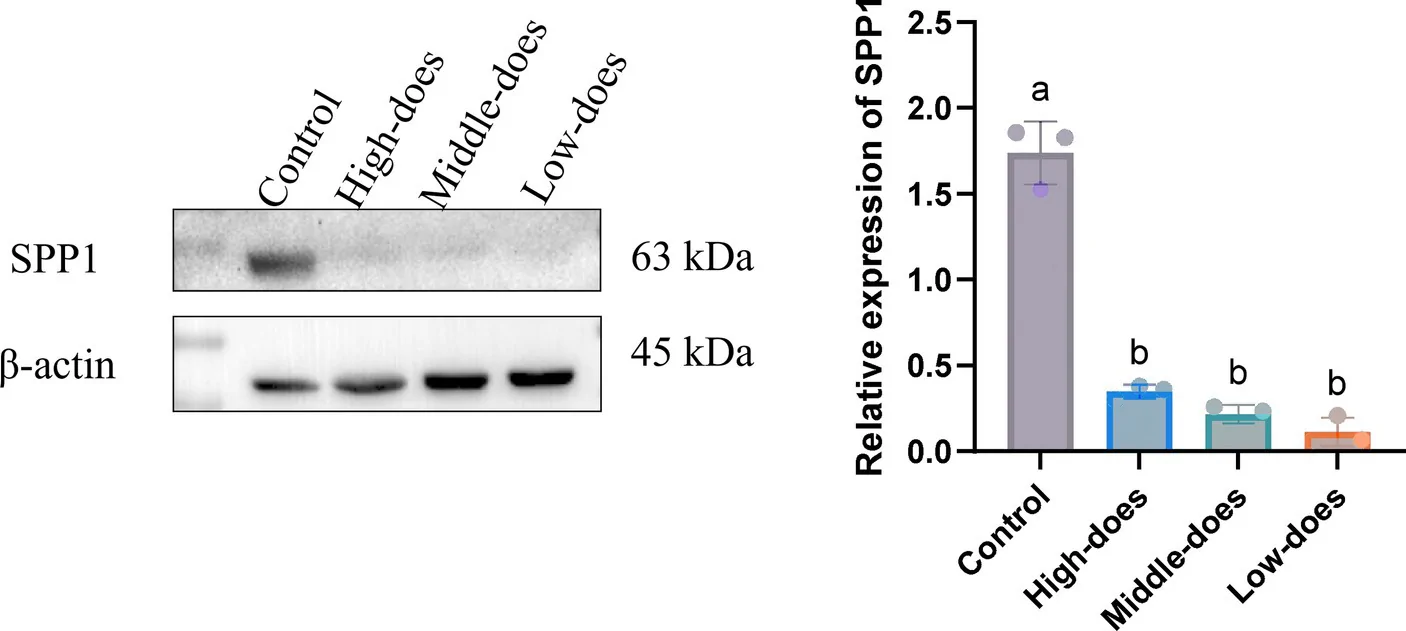
Validation of the effect of H1N1 infection on SPP1 protein expression in piglet lungs by Western blot analysis.

The findings (Figure 8) revealed that, compared with the control group, the protein expression levels of SPP1 in the lungs of piglets in all H1N1-infected groups were significantly decreased (*p* < 0.05).

## 4 Discussion

In the current study, bioinformatics and machine learning approaches were integrated to accurately identify the core host target gene SPP1 from transcriptomic data of H1N1-infected piglets. Furthermore, its expression changes were validated at the protein level using piglet models infected with different viral doses. These findings provide novel targets and theoretical insights for elucidating the molecular mechanisms underlying porcine H1N1 infection.

### 4.1 Advantages and reliability of the integrated multi-machine learning screening strategy

In this study, four machine learning algorithms, including LASSO regression, random forest, SVM, and XGBoost, were innovatively integrated to perform multidimensional feature screening through cross-validation and feature importance ranking, and the intersection of the different algorithms was subsequently used to identify the core target gene. Among these methods, LASSO regression achieves feature sparsity through L1 regularization and effectively addresses the collinearity problem in high-dimensional datasets (9). Random forest, based on an ensemble learning strategy, reduces the risk of overfitting through the voting mechanism of multiple decision trees (10). SVM-recursive feature elimination (SVM-RFE) progressively optimizes the feature subset through recursive elimination procedures (11), whereas XGBoost enhances model generalization ability through gradient boosting combined with regularization terms (10).

Because these four algorithms are based on distinct theoretical principles and possess complementary advantages, the commonly identified gene, SPP1, demonstrated high robustness and reliability. Notably, the AUC values of the four machine learning models all greater than 0.9, indicating excellent discriminative performance of the classification models constructed based on differentially expressed genes. In contrast, the logistic regression model exhibited poor predictive performance (AUC = 0.536), further highlighting the superiority of nonlinear models when dealing with complex high-dimensional datasets. Collectively, this “multi-algorithm cross-validation” strategy effectively overcomes the limitations of individual methods and substantially improves the accuracy and reliability of target gene screening.

### 4.2 Biological functions of SPP1 and its potential role in influenza virus infection

SPP1 (Secreted Phosphoprotein 1), also known as osteopontin (OPN), is a highly phosphorylated glycoprotein rich in aspartic acid residues that is mainly localized in the extracellular space and endoplasmic reticulum. It participates in multiple pathophysiological processes through autocrine and paracrine mechanisms (9, 12). Previous studies have demonstrated that SPP1 plays important roles in immune regulation, inflammatory responses, tissue repair, and apoptosis (13, 14). In the context of viral infections, SPP1 has been reported to participate in infections caused by multiple pathogens, including HIV, HBV, and hepatitis C virus, where it exerts antiviral effects by regulating macrophage polarization, promoting Th1 immune responses, and enhancing natural killer (NK) cell activity (15, 16).

A growing body of evidence suggests that pathogen infection can lead to reduced host SPP1 expression, which is closely associated with the establishment of an immunosuppressive state. Quantitative proteomic analyses have shown that infection of macrophages with *Streptococcus suis* serotype 2 significantly downregulated host SPP1 expression at the transcriptional level, accompanied by coordinated suppression of immune-related genes such as Src, Clec4e, and Mcl1, suggesting that the pathogen may actively manipulate host transcriptional programs through virulence factors such as MetQ (17). In addition, immunosuppressive cytokines, including IL-10 and TGF-*β*, can negatively regulate SPP1 expression through the STAT3/Smad signaling pathway during the late stage of infection (14). Metabolic reprogramming of immune cells from glycolysis toward oxidative phosphorylation, accompanied by M2 macrophage polarization, also contributes to the reduction of SPP1 expression, which is considered a marker of M1 macrophages (18). Furthermore, epigenetic modifications, such as DNA methylation of the SPP1 promoter and histone deacetylation, may lead to long-term silencing of SPP1 expression (14). Therefore, downregulation of SPP1 may not only reflect immune exhaustion caused by immunosuppression but may also further impair antigen presentation efficiency and promote macrophage polarization toward the M2 phenotype, thereby facilitating persistent infection (15).

In this investigation, GO and KEGG enrichment analyses revealed that the differentially expressed genes were mainly enriched in immune response, inflammatory regulation, and cytokine–cytokine receptor interaction pathways, which is highly consistent with the known biological functions of SPP1. As an important immunoregulatory molecule, SPP1 can interact with integrin receptors, such as *α*vβ3 and α4β1, as well as CD44 (12, 19, 20), thereby activating downstream signaling pathways including PI3K–Akt and MAPK (14, 21, 22). These signaling cascades subsequently regulate the secretion of inflammatory cytokines, such as IL-6 and TNF-α, and modulate macrophage activation status (19).

We integrated two public transcriptomic datasets GSE40092 and GSE28871 covering four distinct H1N1 strains to screen host candidate genes, and further performed *in vivo* validation using piglet models infected with the separate H1N1 strain CVCC AV1522. Significant differential expression of SPP1 was detected across all three sample cohorts exposed to different H1N1 isolates. The consistent expression pattern across multiple datasets indicates that altered SPP1 levels constitute a universal host response to H1N1 influenza, rather than a transcriptional change unique to a single viral strain. Such steady differential expression across diverse viral backgrounds strengthens the reliability of SPP1 as a shared marker for H1N1 infection. Our experimental evidence only confirms the significant downregulation of SPP1 expression during the late stage of H1N1 infection. Combined with the above published literature, we put forward several tentative mechanistic speculations that require further experimental validation. On the one hand, the decreased abundance of SPP1 may imply weakened pulmonary innate immune responses and impaired inflammatory regulatory capacities, which could potentially reduce viral clearance efficiency and sustain progressive tissue damage. On the other hand, suppressed SPP1-dependent tissue repair and epithelial regeneration might delay the reconstruction of alveolar structures, which may partially provide a speculative explanation for the prolonged recovery of lung function frequently observed after influenza virus infection. Moreover, reduced SPP1 expression is hypothesized to impair antigen presentation by dendritic cells as well as T-cell activation and proliferation, which might weaken the initiation and maintenance of adaptive immune responses (15). Collectively, we tentatively infer that these molecular alterations may contribute to a potential vicious cycle characterized by “immunosuppression–persistent viral replication–aggravated tissue injury.”

There are two objective constraints in this study. First, only three pigs were used in each of the four experimental groups, resulting in a limited sample size. Second, samples were only collected at day 8 post-infection without serial time-point data across the whole disease course. Besides, we only observed the downregulation of SPP1 upon H1N1 infection, and the relevant immune regulatory mechanisms are merely hypotheses deduced from published literature. In follow-up work, we will expand animal sample size, set multiple sequential sampling time points, and establish cellular and animal intervention models to explore the potential regulatory effects of SPP1 on macrophage polarization, pulmonary inflammatory injury and downstream signaling pathways, so as to further verify the conjectures proposed in this study.

## 5 Conclusion

In this study, we combined bioinformatics and machine learning analysis with piglet models challenged by different doses of H1N1 virus, and identified SPP1 as a host gene with altered expression during porcine H1N1 infection. The observed downregulation of SPP1 at day 8 post-infection may potentially correlate with attenuated pulmonary innate immunity and compromised tissue repair capacity at the late infection phase. Collectively, these findings supply preliminary data to support subsequent exploration of the molecular regulatory network of porcine H1N1 infection, and may aid the screening of candidate molecular targets for influenza prevention and treatment.

## Data Availability

The original contributions presented in the study are included in the article/supplementary material, further inquiries can be directed to the corresponding author.
